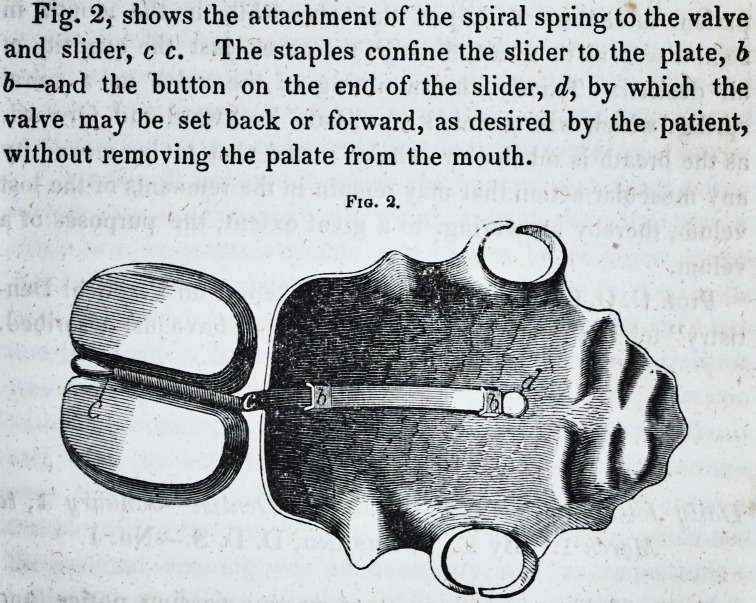# A New Kind of Artificial Palate: With Observations on the Changes That Take Place in the Velum and Pharynx, during Deglutition, Respiration and Articulation

**Published:** 1851-04

**Authors:** S. P. Hullihen

**Affiliations:** Wheeling, Va.


### ARTICLE III.

*A New Kind of Artificial Palate: with Observations on the Changes that take place in the Velum and Pharynx, during Deglutition, Respiration and Articulation.* By DR. S. P. HULLIHEN, of Wheeling, Va.

ARTIFICIAL teeth, legs and eyes, are the only portions of the human body that are usually supplied with any degree of ornament or usefulness; and the beauty and usefulness of these depend upon the *amount* of motion that is capable of being imparted to the movement of the artificial parts. Hence, artificial teeth stand preeminent: the motion of the jaws being in no way impaired in the loss of the natural teeth. Artificial ones can, therefore, be endowed with almost the same beauty and usefulness with which natural ones are. The usefulness of the artificial leg is, more or less, depending entirely upon the amount of muscular power or motion that may be imparted to it, from the remnant of limb on which it is attached. The natural appearance of the artificial eye depends as much upon the motion that is given to it, as upon either its size or color. Without procuring motion, therefore, from the neighboring parts, or rather from the muscles concerned in *some* of the movements of the lost parts, artificial substitutes will be devoid of either usefulness or ornament. It is upon this principle, that the artificial palate, shortly to be described, was planned.

Before entering, however, upon the description of this kind of palate, it may not be amiss, first, to refer to some actual observations made upon the movements which occur in the velum or soft palate and pharynx, during the performance of deglutition, respiration and articulation—for the purpose of learning, if possible, how far the offices of the velum may be supplied by mechanical means.

In the year 1837, Dr. Hilton, of England, removed a large bony tumor from the face of a patient, aged about thirty-six years. The chasm left by this operation "was bounded *below*

by the nasal surface of the hard palate and the floor of the left antrum; *above*, by the left frontal sinus and left half of the cribriform plate of the ethmoid bone; *internally*, by the septum nasi, which presented a general concave surface, with a small opening through it at the lower part, communicating with the left nostril; and *externally*, by the left orbit; *posteriorly*, it opened into the pharynx,"—exposing to perfect view the following very interesting physiological facts.

"*Respiration.*—During quiet and unconstrained breathing, *with the lips closed*, so that the air must pass through the nose and opening, there is not any perceptible movement upwards of the soft palate; nor does the pharynx advance. The latter remains perfectly still; and the former, we may conclude, as it disappears in the descending direction, is closely adapted to the back part of the mouth, so as to leave, for the air passing to and from the larynx, a free and spacious canal.

"When *the lips are slightly separated*, so that the respiration is in part carried on through the mouth, the soft palate is seen to be carried, or rather, perhaps, drawn, upwards and backwards, at each inspiration, and at each expiration again to decline; the pharynx continuing almost at rest, but having a slight disposition to advance.

"Upon taking a full inspiration through the mouth, the palate is directed more completely upwards and backwards, and adapts itself to the advancing pharynx. This adaptation remains until the expiration has nearly terminated; but it should be remarked that the sides of the pharynx do not, even in this case, approximate so much as during deglutition.

"In smelling, there is no perceptible movement of the palate nor of the walls of the pharynx.

"In holding the breath, as it is termed, or forcibly retaining the air in the chest, either with the lips closed and the tongue depressed, or with the tongue raised and adapted to the concave surface of the hard palate, and the lips separated—or, lastly, with the superior and posterior portion of the tongue applied, by great voluntary effort, to the concave commencement of the soft palate, the lower jaw being depressed, and carrying with it the genial portions of the genio-hyo-glossi muscles—the pharynx advances, and the palate is much raised, presenting a convex surface upwards, undulating, with a somewhat tremulous motion, upon the subjacent column of air."

Mr. Hilton observes, that "the closure of the glottis alone, can retain the air within the chest but for a very short time, and that the assistance of the soft palate, as well as of the tongue and lips and jaws, is highly useful."

"Sneezing consists of inspiration through the mouth when the palate is raised, and of expiration through the nose when the palate is depressed. But if sneezing happens many times in quick succession, so that the palate has scarcely time, after its elevation during the oral inspiration, to descend to the back of the mouth, before the air returns from the chest, the palate is then seen violently agitated by the air in its ascending direction, being at that time obliquely placed with respect to the expired air, so that a portion of the air passes through the nose, and a portion through the mouth, giving the coarse, tearing sensation to the palate, and flapping motion of the lips, expeperienced in the imperfect attempt to sneeze."

"*Deglutition.*—When the mouth is open to receive the food, the palate is raised, but not so completely as in full oral inspiration; the sides of the pharynx also approximate, but not so closely as on drawing the breath inwards; the posterior part of the pharynx advances but slightly. Directly the food, either solid or fluid, is placed in the mouth, the palate descends, and continues, during the detention of the food, closely adjusted to the back part of the mouth, the pharynx remaining perfectly quiet. These conditions are to be observed during the process of mastication, performed with or without food in the mouth. Extreme lateral movements of the jaw encroach upon the pharynx but slightly; and in the ordinary lateral motion there is no difference to be observed in its capacity. Immediately antecedent to that part of the process of deglutition occurring in the passage of the food between the fauces and pharynx, or when the food is passing backwards over the upper opening of the larynx, the palate is carried *completely* upwards and backwards, and the pharynx advances, the sides of which, more especially, approximate. At this time the thyroid cartilage rises. These movements of the larynx and palate occur nearly simultaneously, those of the palate having but a momentary precedence. The palate and pharynx being now nicely adjusted, their common surface presents, from above, a hollow cone, in consequence of the partial descent of the palate; but so closely is their adaptation maintained during the deglutition, that not the slightest portion of the passing substance is perceived above the palate. At this moment the pharynx is indeed divided into two distinct cavities—one at the upper part, common to the nose and pharynx—the other open to all the apertures below the palate. As the fluid or food passes into the lower part of the pharynx, the upper portion recedes or retires from the palate, the soft palate falls, and last in this succession of events, the larynx, as is indicated by the motion of the thyroid cartilage, descends." By these observations, we learn that the velum has many of the characteristics of a valve.

"*Articulation.*"—On this head, Dr. Hilton's observations were unsatisfactory; indeed, it could not be otherwise—the eccentric movements of the soft palate—the difference in its movements in uttering the same words in different tones of voice—the frequent sameness of its position during the delivery of entire sentences, and its wild tumult in others, render a description of its movements during articulation beyond the power of description. Upon this point I speak with some experience, having had a case, about a year since in which the movements of the palate were clearly visible during articulation, yet I was unable to make any observations that were in any degree perfect or worthy of record. But, however interesting such facts would be in a physiological point of view, to know every change that takes place in the soft palate during articulation, would be of no practical importance in the construction of an artificial one. The beautiful and perfect mechanism of the velum and pharynx, described by Dr. Hilton—their uniform and associated movements during respiration and deglutition, shadow forth, most clearly, that an artificial substitute, so contrived as to fulfil, so far as possible, the indications of a velum, and so arranged in the mouth that it would receive muscular motion from whatever remnants may remain of the destroyed velum, would accomplish all that art could do, even were it possible to be in possession of every *quiver* of the velum, that may take place in giving utterance to every word in every living language. I say, "from whatever remnants may remain of the destroyed velum," for no one, I presume, having the least claim as a dental surgeon, would be inconsiderate enough to attempt the application of an artificial substitute in the case of a cleft velum or soft palate, with any hope of success. In such cases staphyloraphy is the only resource, and no one, I will venture to predict, will scarcely ever see a case, where the velum has been destroyed by disease, in which there will not be remnants enough, with muscular power enough, to work an artificial substitute with more or less benefit to the patient, if properly applied.

There is a great difference, however, attending the success of improving the voice, in patients where the velum has been lost by disease, and where the palates are cleft. Patients who have once acquired a correct enunciation, and lost those parts by disease, note with horror the change that takes place in their voices, and experience difficulties that are grievous to be borne, in their articulation, and these defects are always present in their minds. In such cases, an artificial substitute, having in view the restoration of the air-passages to their normal condition, will at once improve the voice and delight the patient. On the other hand, patients having cleft palates do not *hear*, or rather they do not note, the defects in their own voices; they likewise have acquired a natural habit of speech, peculiar to that class of patients, and any change made in the deficient parts, they almost feel to be an impediment, rather than a benefit. In fact, they do not know how to make a correct articulate sound, and, indeed, they can scarcely ever be taught to do so, even when their palates have been perfectly restored, unless that restoration is effected at an early period of life. Therefore, defects of the voice, attending cleft palates, may be sometimes remedied by surgical means—but by mechanical means—never.

But it is not my intention at this time, to treat of the uses and abuses of artificial palates, nor to give anything like a history of all the modifications that may be required in different cases. I only propose to describe *a kind of valve*, which may be attached to any plate, of any desired size or shape—a valve that may be set while in the patient's mouth, in an instant, so as to admit, through the nares, just the volume of air desired—and one that may have many movements of the velum, being impelled by whatever remnants of muscles may remain of that organ.

An artificial palate made upon this plan, will be composed of four parts: 1st. A valve, made from gold plate, as thin as it can well be worked; 2d. A spiral spring, about an inch long, and of the size usually made for whole sets of teeth; 3d. A slider, one inch and a half in length, and of the width and thickness of a common watch-spring; 4th. A plate, larger or smaller, as the case may require, struck up in the usual way, to fit the roof of the mouth.

The size and form of the valve is obtained by taking an impression of the posterior opening of the nares: the plate composing it should be struck up in two parts, front and back, which, when soldered together, makes a hollow body, of the form in Fig. 1, letter *a*. At the upper end of the valve, a small pin is soldered, the point of which looks downwards, and of sufficient thickness to fit very tightly in one end of the spiral spring. The spiral spring must be made of such a length as will permit the valve to rest slightly upon the upper surface of the remnants of the lost velum. The slider has a pin in the posterior end, looking upward to receive the other end of the spiral spring before described. The anterior end of the slider has a small button looking downward; the slider is attached to the plate by two small staples, as represented in Fig. 2, *b b*. The plate may be made to cover the entire roof of the mouth when necessary; or it may be made only sufficiently large to permit the mounting of the slider. These different parts, when put together, particularly if the plate is to cover the whole roof of the mouth, makes a palate of the form represented by Fig. 1.

FIG. 1.

Fig. 2, shows the attachment of the spiral spring to the valve and slider, *c c.* The staples confine the slider to the plate, *b b*—and the button on the end of the slider, *d*, by which the valve may be set back or forward, as desired by the patient, without removing the palate from the mouth.

FIG. 2.

The palate should be made to fit the several parts for which it is intended, with great exactness. The plate must fit the roof of the mouth, and the teeth to which it may be secured, in a faultless manner. The slider must be arranged so as to permit the valve to be drawn so closely against the posterior opening of the nares, as to close them; or to be pushed back so as to leave them entirely unobstructed. The spiral spring, as I have before remarked, must be made of such a length as will allow the valve to rest slightly upon the upper surface of the remnants of the lost velum. The valve should be sufficiently wide at its base, to overlap the remnants of the velum, so far as the parts on each side will permit, without producing irritation—any other part of the valve than the base, should not be allowed to touch, unless when brought forward against the nares. Unless all the parts are so arranged, the palate will not be properly constructed, and will not of course, answer the end desired.

Thus it will be perceived, that the peculiarities of this palate, are—First, a valve made to fit the posterior opening of the nares. Secondly, the attachment of this valve to a slider, by which the patient is enabled to adjust the valve while in the mouth, in such a way as to admit through the nares, just the quantity of air desired. Thirdly, the mounting of the valve on a spiral spring, which will permit it to vibrate backward and forward, as the breath is inhaled or exhaled; and also to be moved by any muscular action that may remain in the remnants of the lost velum, thereby answering, to a great extent, the purposes of a velum.

Prof. C. O. Cone, in his very able "Report on Practical Dentistry" for 1848, refers briefly to the palate I have just described.

### ARTICLE IV.

*Daily Jottings in the Gold Book of a Dentist—January* 1, *to March* 1. By E. TOWNSEND, D. D. S.—No. 1.

## Figures and Tables

**Fig. 1. f1:**
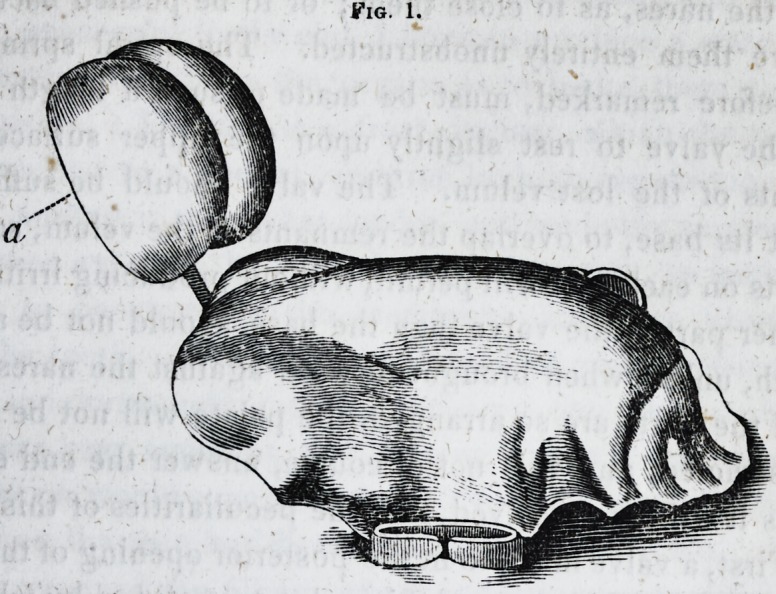


**Fig. 2. f2:**